# Facilitating reproducible research through direct connection of data analysis with manuscript preparation: StatTag for connecting statistical software to Microsoft Word

**DOI:** 10.1093/jamiaopen/ooaa043

**Published:** 2020-11-06

**Authors:** Leah J Welty, Luke V Rasmussen, Abigail S Baldridge, Eric W Whitley

**Affiliations:** o1 Division of Biostatistics, Department of Preventive Medicine, Northwestern University Feinberg School of Medicine, Chicago, Illinois, USA; o2 Division of Health and Biomedical Informatics, Department of Preventive Medicine, Northwestern University Feinberg School of Medicine, Chicago, Illinois, USA; o3 Department of Preventive Medicine, Northwestern University Feinberg School of Medicine, Chicago, Illinois, USA

**Keywords:** dynamic documents, reproducible research, open science, software

## Abstract

**Objectives:**

To enhance reproducible research by creating a broadly accessible, free, open-source software tool for connecting Microsoft Word to statistical programs (R/R Markdown, Python, SAS, Stata) so that results may be automatically updated in a manuscript.

**Materials and Methods:**

We developed StatTag for Windows as a Microsoft Word plug-in using C# and for macOS as a native application using Objective-C. Source code is available under the MIT license at https://github.com/stattag.

**Results:**

StatTag links analysis file(s) (R/R Markdown, SAS, Stata, or Python) and a Word document, invokes the statistical program(s) to obtain results, and embeds selected output in the document. StatTag can accommodate multiple statistical programs with a single document and features an interface to view, edit, and rerun statistical code directly from Word.

**Discussion and Conclusion:**

StatTag may facilitate reproducibility within increasingly multidisciplinary research teams, improve research transparency through review and publication, and complement data-sharing initiatives.

**LAY SUMMARY**

Investigators preparing manuscripts often transcribe results from statistical analyses into Microsoft Word documents. The challenge with this process is that it is potentially error prone, and provides no record of the link between a number in the manuscript and the data or analysis that created it. We developed StatTag to address this problem. StatTag is a free and open-source program that connects Microsoft Word to popular data analysis programs: R/R Markdown, Python, SAS, and Stata. Using StatTag, investigators can automatically update the results in a manuscript when data or models change and can inspect the statistical code that created every result. StatTag provides an additional tool for investigators to make their research more efficient, robust, and reproducible.

## INTRODUCTION

Conducting reproducible research requires that data generation and analysis be sufficiently documented so that results can be recomputed or verified.^1–6^ There are many important aspects to reproducibility,^6^ one of which is that every result in a manuscript should be readily traceable to the data and the statistical analysis that produced it.^7^ One approach to this aspect of reproducibility is “weaving” manuscript text and statistical analysis code together into 1 file, a practice originally coined as “literate programming.”^8^ This requires using a plain text editor to intersperse “chunks” of statistical code with manuscript text and using a markup language to indicate formatting. When this file is compiled, the statistical results are automatically updated and embedded in the formatted manuscript—the “dynamic document”—generated as a PDF, HTML, or DOCX file. Weaving prevents transcription errors, and the source file provides a single location identifying the data, analytic methods, and numeric results, along with their interpretation. Some of the first weaving programs include Sweave and SAS’s Output Delivery System.^9^^,^^10^ Tools have evolved rapidly over the past 15 years, especially for R statistical software. The most popular weaving tools today include R Markdown and knitR.^11^^,^^12^ A more recent system, Manubot, uses GitHub, shell scripts, and configuration files to weave templated markdown documents with the results generated.^13^

Although weaving tools have transformed the practice of reproducible data analysis, investigators often transcribe results into Microsoft Word documents, for example, when preparing manuscripts for submission. Both the authors’ experiences and related survey data suggest that weaving tools are not widely used throughout medical and scientific research.^1^ Multiple factors may contribute to limited uptake. First, the technical skills to write and compile documents in plain text editors may create a barrier to entry (eg, both Sweave and knitR require LaTeX,^14^ a typesetting system often used for formatting mathematical documents). Second, authors may prefer Word’s “what you see is what you get” interface and editing features that encourage collaborative, visual editing with real-time formatting. In contrast, “weaving” tools portray plain text interspersed with statistical code such that the formatted appearance is not clear until the document is compiled and rendered; user interfaces for comment and review are limited. Third, weaving tools are often designed to work with a single statistical program and cannot accommodate analyses completed with multiple software packages. Fourth, the robustness, support, and knowledge base for weaving tools varies across statistical packages. As of this writing, R users have a rich set of weaving tools in R Markdown and knitR, which are developed and supported by an open-source community. In the authors’ experience, however, SAS and Stata users have more limited and sometimes less robust options, with fewer extensions and customizations provided by the vendors or a broader open-source community. Finally, although some weaving tools can generate Word documents, to maintain reproducibility, any changes must be reentered in the plain text source file—an untenable process for many nontechnical collaborators.

In contrast to weaving, Word is ubiquitous for manuscript preparation for medical and scientific journals, but does not itself facilitate reproducibility. Both *JAMIA Open* and *JAMIA*, as well as many other well-known journals prefer or require that manuscripts be submitted as Word documents.^15–21^ Authors therefore frequently transcribe results into Word documents, severing provenance to the data and analyses and potentially introducing errors. Weaving tools are of limited benefit when drafting manuscripts in Word: the final step of copying-and-pasting breaks a link in the reproducibility chain.

With this challenge in mind, we developed a broadly accessible, free, open-source software tool, StatTag,^22^ to support reproducible research by connecting data analysis with manuscript preparation in Word. In what follows, we describe the development approach, features, and software architecture as well as how to obtain and use StatTag. We conclude with a discussion of future directions, planned enhancements, and the role of programs like StatTag in supporting one aspect of reproducibility in an increasingly multidisciplinary research environment.

## METHODS

### System design

We developed StatTag collaboratively as a team consisting of 2 biostatisticians and 2 software developers, starting in October 2015. Based on our collective experiences as part of multidisciplinary teams engaging in clinical and translational research, we established 5 primary goals for StatTag: (1) minimize technical and nontechnical barriers to adoption; (2) support multiple statistical programs, even within the same Word document; (3) leverage native Word features, including “track changes” and formatting; (4) allow a bidirectional workflow (eg, inserting statistical output in Word plus editing and running code files from Word); and (5) interface seamlessly across collaborators, even if some do not have StatTag or the statistical code.

We conceptualized StatTag for the Windows operating system as an “add-in” to Word, integrating StatTag within the Word toolbar. StatTag for macOS was developed as a standalone application because of how macOS implements Word and application interoperations. We designed StatTag for versions of Microsoft Word and statistical software running locally on a desktop or laptop. The online version of Office 365 does not interoperate with local statistical programs, and statistical programs accessing sensitive or protected information are frequently run locally rather than in cloud-based environments.

During the iterative development process, we gathered feedback from informal key informant interviews, focus group discussions, surveys, and workshops with unpaid volunteers who we considered to be potential users. Given that these activities were not intended for research purposes, informed consent was not obtained, and exemption for survey administration was provided by the Northwestern University Institutional Review Board.

### Implementation

**Figure 1.**
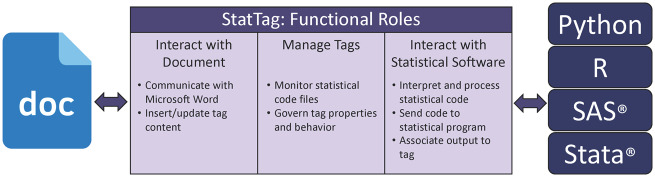
Schematic overview of the 3 primary functional roles implemented within StatTag and the responsibilities of each role.

StatTag is implemented with 3 primary functional roles (Figure 1). The first manages the interaction with Word, including communications with the Word document to insert and update the statistical output. Values and table data are inserted as “field codes,” an approach inspired by the Stata Automation Report Project.^23^ Figures are inserted as images, and verbatim output is inserted as a text box. The second functional role is managing the statistical output to be inserted into the Word document. StatTag maps statistical output (tags) to Word content, including tag names, their locations in the Word document, what statistical code they come from, and how they should be formatted. The third functional role is to connect to the statistical application. In this process, StatTag reads and preprocesses the code file(s) in the order they have been linked to the Word document. Preprocessing includes breaking the statistical code into a collection of code blocks and performing normalization specific to the statistical program. For example, a Stata command that uses “\” to span multiple lines will be collapsed into a single line to send to Stata. StatTag then sends the code to the appropriate statistical program using the program’s Application Programming Interface (API). When invoking the API, StatTag uses the default environment available for the statistical program, and all dependencies are expected to be managed by the statistical program itself (eg, installing R packages). StatTag runs R, Python, SAS, and Stata code in “batch mode;” code that runs in the statistical application should also work with StatTag. Finally, StatTag associates the resulting statistical output with tags so that they may be inserted into the Word document.

**Figure 2.**
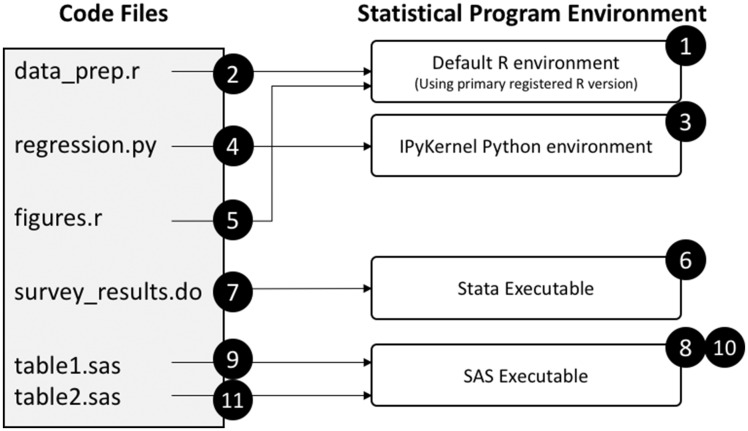
StatTag’s execution process of multiple linked code files in a single Word document. The code files are listed in the order they were linked to the Word document, which dictates the execution order.

A description of the execution steps for an example set of code files is illustrated in Figure 2 and described below. We included all file types (R, Python, Stata, and SAS), as well as 2 separate R files and 2 separate SAS files to demonstrate how files of the same type are run.

1. StatTag initializes an R environment to use for the data_prep.r file. Using the system settings, StatTag finds the active R version, as determined by registry keys in Windows or by dynamic library registration on macOS, and creates an embedded R environment.
2. In a preprocessing step, StatTag creates “code chunks” as either the block of code outside of a tag, or all of the code within a tag. StatTag executes the code in the R file by sequentially sending chunks of code to be run within R. Results from tagged code chunks are saved for later document updates. Because of how the R.NET library manages the R environment, R is not shut down after all the code chunks are run. This is different behavior than for the other statistical programs.
3. StatTag initializes a Python environment for the regression.py file. Because StatTag uses Jupyter for Python integration, it invokes the Python environment registered with the IPyKernel.
4. StatTag executes the code in the Python file. Using the Jupyter protocol, it sends User Datagram Protocol messages to the IPyKernel, which in turn sends code chunks to be executed in the Python runtime. Unlike R, at the end of the Python file, StatTag shuts down the Python kernel.
5. StatTag is now ready to run figures.r. Because StatTag did not shut down the R environment at the end of Step 2, it has access to the same R instance (and environment). The code in figures.r is then executed.
6. StatTag initializes the Stata runtime (via the Stata Automation API), which launches the Stata executable. The version of Stata run depends on which version of the Stata Automation API is registered by the user on their system.
7. StatTag executes the Stata code, sending code chunks to the Stata environment to be run. At the end of the execution of this code file, the Stata environment is shut down.
8. StatTag initializes the SAS executable on the system, using the default registered version of SAS.
9. StatTag executes the code by sending code chunks from the table1.sas file. At the end of the code file, StatTag closes the SAS session.
10. StatTag now has another SAS file to run (table2.sas). Because it shut down the SAS environment at the end of Step 9, it now has to initialize the SAS executable again using the same process as in Step 8.
11. StatTag executes table2.sas, and once again shuts down the SAS environment when it is complete.

StatTag interfaces independently with R/R Markdown, Stata, Python, and SAS (Windows only; there is no native version of SAS for macOS). Because of this approach, users or development teams may readily incorporate new statistical programs or programming languages. For example, future versions of StatTag may work with MATLAB or SQL. Server-based software programs are not currently supported as StatTag relies on APIs for interoperability with statistical programs.

Table 1 summarizes the design and implementation of both the Windows and macOS versions. The Windows and macOS versions of StatTag intentionally share the same functionality but deliver specialized, platform-specific user applications that are tailored to each operating system and its established conventions. Both applications are available for download at the StatTag website (stattag.org); source code is available on GitHub (github.com/StatTag).

**Table 1.** Design and implementation of StatTag for Windows and macOS

	Windows	macOS
Application interaction	“Add-in” to Microsoft Word	Standalone application
Development language	C#	Objective-C
Frameworks	Microsoft.NET, Windows Forms,^24^ Component Object Model	Cocoa and AppleScript
Supported statistical software	R, R Markdown, SAS, Stata, Python	R, R Markdown, Stata
Application availability	Free download at stattag.org	Free download at stattag.org
Source code	https://github.com/StatTag/StatTag	https://github.com/StatTag/StatTagMac
License	MIT	MIT

### Software use

The Windows and macOS versions are used identically though they differ visually and have slightly different features. The main steps in using StatTag are shown in Table 2, with illustration and further description in Supplementary Figure S1. Additional resources, including the User’s Guide, instructional videos, and an online short course, are available at the StatTag website. StatTag v5.0.2 was used to insert the usage numbers presented in the “Results” section.^22^

**Table 2.** Steps for working with StatTag to collaboratively prepare a document

Step	Process	Description
1	Developing statistical code	Using text editor of choice, write statistical code in R/R Markdown, SAS, Stata, and/or Python. Code files should run without error before use with StatTag
2	Associating a code file	Using StatTag, associate one or more code files with the Word document
3	Defining tags (tagging)	Using StatTag, identify results (numeric value, table, figure, or verbatim output) within a code file to embed within the Word document or Using text editor of choice, identify results (numeric value, table figure or verbatim output) within the code file using specially formatted comments
4	Inserting tags	Using StatTag, insert tags into user defined locations in the Word document. Tags that are copied and pasted retain their links to the statistical code and will update accordingly
5	Working collaboratively	Working in Word, share and edit document with collaborators as needed, using track changes and comments. Collaborators wishing to only modify the Word document do not need to have StatTag installed. Any collaborator with access to the source code and data can add or update tags using the exact same process
6	Updating results	Using StatTag, update results within the Word document

## RESULTS

**Figure 3.**
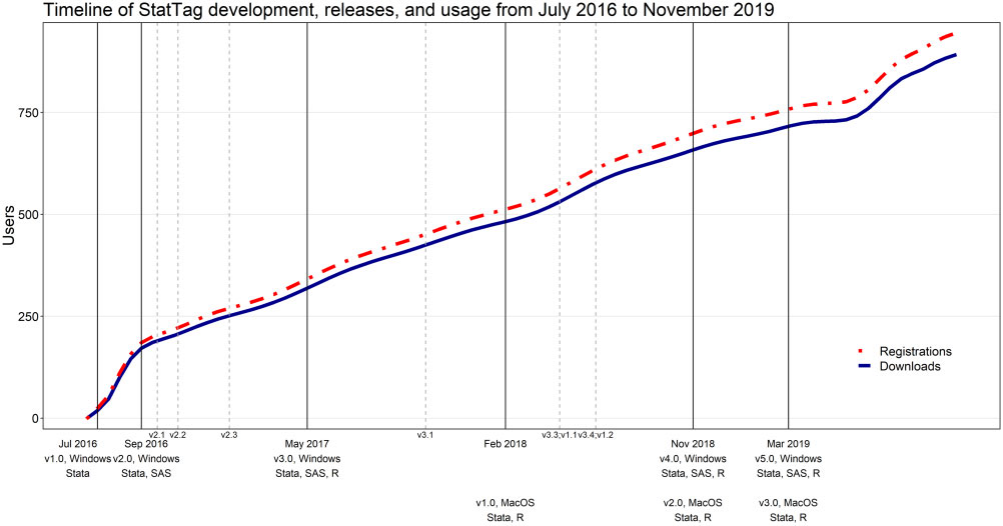
Timeline of StatTag development, releases, and usage from July 2016 to November 2019. The number of registrations at stattag.org are shown in red; the number of registrations that also included a download of StatTag are shown in blue. Solid vertical lines indicate “major” releases, such as addition of a statistical software program or substantial redesign of the user interface. Dashed vertical lines indicate “minor” releases, which include smaller changes such as improving speed or addressing user-reported issues.

As of May 2020, the most current versions of StatTag were v6.0 for Windows (64-bit or 32-bit) and v3.0 for macOS (Figure 3). The project website (stattag.org) contains a comprehensive list of releases and development versions. Through November 1, 2019, users were asked to create a secure account prior to download as well as provide optional demographic information. As of November 2019, 943 users affiliated with more than 244 institutions had registered to download StatTag. They self-reported affiliation with a wide variety of nonexclusive fields, including 22% clinical medicine, 26% public health, 13% behavioral science, 41% biostatistics, 25% epidemiology, 16% economics, and 19% mathematics.

During the design and development process, key informant interviews, focus groups, surveys, and workshops resulted in 372.5 contact hours with at least 203 individuals, including graduate students (statistics and biostatistics) and professionals in academia, industry, and government who had some analytic or informatics background. Information gleaned from these activities resulted in alterations to design, additional features, and bug fixes. For example, StatTag had separate screens to add and remove tags, to insert tags, and to update the results. Feedback from users resulted in a combined “tag manager” dialog box with all 3 functions. Additional examples are listed in Supplementary Table S1.

## DISCUSSION

With increasingly multidisciplinary research teams, tools such as StatTag facilitate reproducibility beyond the statistics community, improve research transparency through review and publication, and complement data-sharing initiatives. Using StatTag eliminates copying-and-pasting statistical results into Word: research teams may work separately on the analysis code and the corresponding manuscript but retain connections between the 2. StatTag may be useful for any investigator who prepares manuscripts that include statistical results: it is designed to be sophisticated enough to appeal to the expert statistician, but accessible enough for scientists and clinicians with limited statistical expertise.

StatTag has the potential to improve research transparency through the review and publication process. Because many journals rely on Word for typesetting and publication, StatTag could be integrated with existing publication workflows. For example, in the Windows version of StatTag, double-clicking on any tagged result in the manuscript opens a dialog box displaying the statistical code that created the result. Editors or reviewers could use this feature to examine analytic code associated with a result as long they had access to the Word document and code. Future versions of StatTag may store read-only copies of code within the document, providing embedded documentation of the analyses.

StatTag does not directly connect to data or store a copy of data in order to avoid inadvertent exposure of protected health information, personally identifying information, or other sensitive information. However, StatTag could be extended with a feature to store a copy of the code and the data, creating a self-contained bundle to dynamically reproduce results in a manuscript. This feature could be enabled to support initiatives to share data from clinical trials or ‘omics studies, for example.^25^^,^^26^

Although StatTag represents a step forward in providing an additional software option to enhance reproducibility, it addresses only one portion of the reproducibility pipeline. StatTag does not manage source control of the statistical code and requires code to be available locally as opposed to reading from an external repository, such as GitHub. In addition, it leverages Word’s “track changes” feature, but does not maintain a history of all changes to the document.

The development and adoption of broadly accessible software tools to facilitate reproducibility throughout the research process is of critical importance. Practicing reproducible research not only increases accuracy and efficiency,^27^ but it also protects investigators and the public from serious consequences. Irreproducibility has led to the retraction of published articles, false leads in preclinical research, incontrovertible damage to careers and professional reputations, diminished confidence in scientific research, and, most seriously within the medical community, potential harm to patients.^28–30^ Many journals recognize the importance of reproducible techniques and now request that authors provide some evidence of a minimum standard of reproducibility before publication.^31–36^ The National Institutes of Health is one among many prominent institutions calling for the development of methods to implement rigor and transparency in research.^37^ Tools like StatTag are critical to meeting demands for scientific transparency and reproducibility, and have the potential to become standard practice for increasingly multidisciplinary research teams.

## FUNDING

StatTag was developed within the Northwestern University Clinical and Translational Sciences Institute, supported in part by the National Institutes of Health’s National Center for Advancing Translational Sciences (grant UL1TR001422).

## AUTHOR CONTRIBUTIONS

All authors contributed to the software design and writing and revising the manuscript. LJW and LVR obtained funding. LVR and EWW wrote the software, and ASB conducted the data analysis.

## SUPPLEMENTARY MATERIAL

Supplementary material is available at *Journal of the American Medical Informatics Association* online.

## Supplementary Material

ooaa043_Supplementary_Data
