# Supplementary material for: Facilitating reproducible research through direct connection of data analysis with manuscript preparation: StatTag for connecting statistical software to Microsoft Word
Source: JAMIA Open. 2020 Nov 6;3(3):342–58. doi: 10.1093/jamiaopen/ooaa043 (PMC7660954; doi:10.1093/jamiaopen/ooaa043)
Supplement: ooaa043_Supplementary_Data [file ooaa043_supplementary_data.docx]

**SUPPLEMENTAL MATERIAL**

**for**

**Facilitating Reproducible Research through Direct Connection of Data Analysis with Manuscript Preparation: StatTag for Connecting Statistical Software to Microsoft Word**

Leah J. Welty, Luke V. Rasmussen, Abigail S. Baldridge, Eric W. Whitley

[S1. Software Usage 2](#_Toc49497521)

[Supplemental Figure 1. 4](#_Toc49497522)

[S2. Interaction with Microsoft Word 5](#_Toc49497523)

[S3. Compatibility and Software Updates 5](#_Toc49497524)

[Supplemental Table 1. 6](#_Toc49497525)

# S1. Software Usage

This section describes the main steps in using StatTag. Additional resources, including an online short course, are available at the StatTag website (stattag.org). **Supplemental Figure 1** illustrates how to use StatTag:

1. *Developing statistical code.* Users write statistical code in their preferred editor. Code files should run without error before use with StatTag. StatTag works with code file(s) written for R (.R) and R Markdown (.Rmd), SAS (.sas), Stata (.do), or Python (.py).
2. *Associating a code file.* From Microsoft Word, the user identifies a code file that should be associated with the Word document. The user may associate more than one code file. StatTag stores the paths to code files within the Word file as a custom document property.
3. *Defining tags (“tagging”).* The next step is for the user to tell StatTag which results from the (now connected) statistical code should be embedded within the Word document. We refer to this process of identifying desired results as defining tags, or “tagging” the code file. Through the graphical user interface (GUI), the user specifies the tag name; which line(s) of the code file create the target output; the type of output (numeric value, table, figure, or verbatim output); and associated formatting (e.g., number of digits for numeric values, which columns if any to exclude for a table). StatTag translates this information into a set of formatted comments, which it inserts into the code file. Of note, advanced users may skip the GUI and instead define tags by writing specifically formatted comments directly in the code.
4. *Inserting tags.* Once a tag is defined, the user then positions the cursor at the desired location in the Word document and “inserts” the tag placeholder. This action inserts the tag name in brackets (e.g. “[my tag name]”) in the Word document. This allows the user to insert multiple tags without running the statistical code file. When finished inserting tags, the user then “updates” selected tags, which runs the statistical code file(s) and fills in placeholders (or updates existing tags) with the results. The StatTag Internals section below describes StatTag’s internal workflow for this process.
5. *Working collaboratively.* The Word document may be shared with collaborators, who may edit the document, including use of Word’s “track changes” feature. StatTag does not currently retain a copy of the statistical code or the data used to generate the results. The statistical code may also be updated in parallel, either using the StatTag interface within Microsoft Word or separately in a text editor or the statistical software. A collaborator with access to the code files and data can add or update tags using the exact same process as the initial author.
6. *Updating results.* Once embedded in the Word document, results can be individually or collectively updated with a behind-the-scenes call to the statistical program(s). The Word document may be edited as usual, and results inserted from StatTag retain their native Word formatting. For example, if a result is bolded in a table, it remains bolded even if the value is updated. In addition, cutting and pasting tagged results within the document retains the links to the statistical code, and results will be updated accordingly when the statistical code is rerun.

Supplemental Figure 1. Screen shots illustrate an example StatTag wokflow for inserting resutls from an analysis in SAS (shown in Step 1) to a formatted table in Microsoft Word (shown in Steps 5 and 6) on a Windows machine.


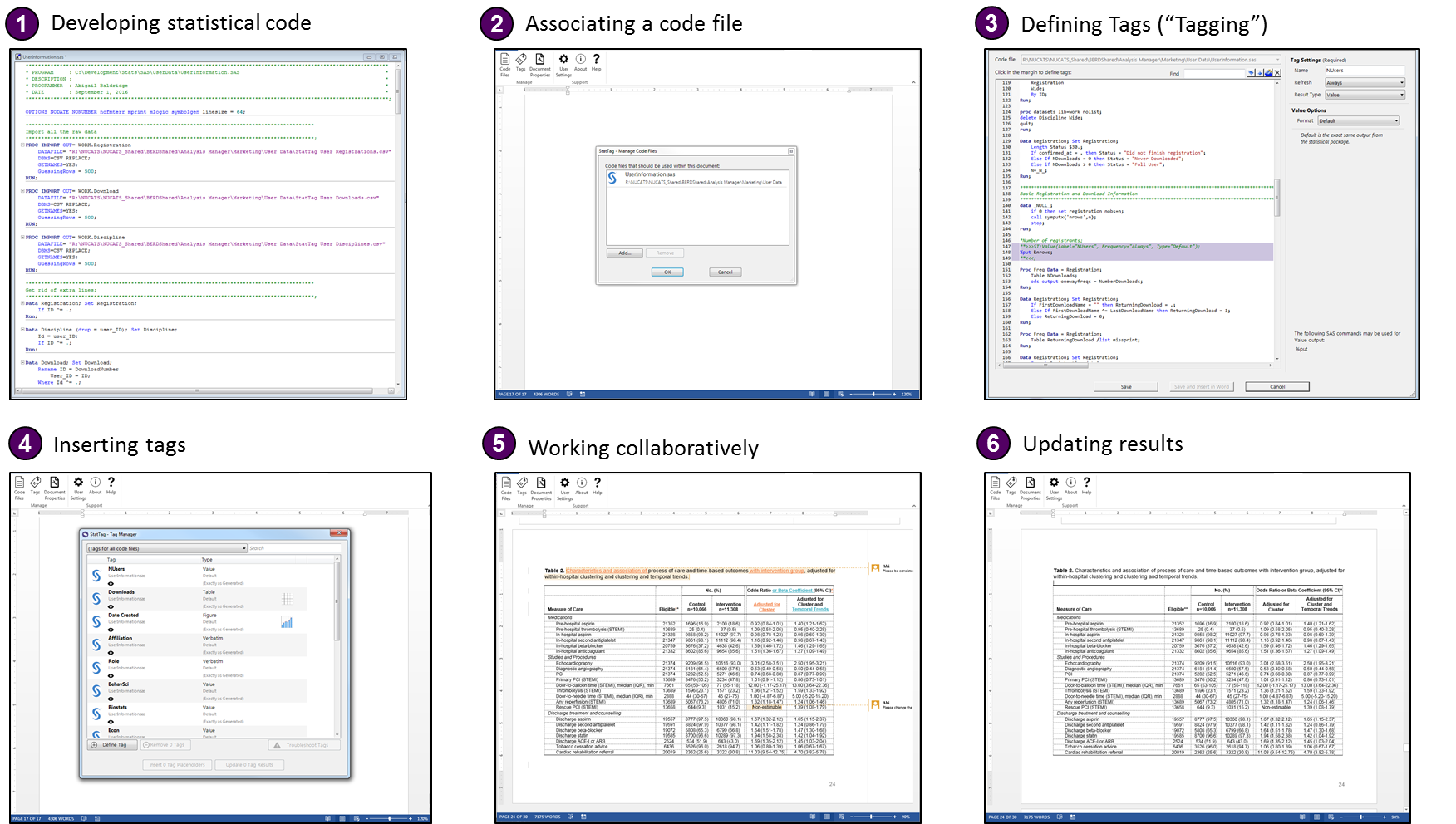


# S2. Interaction with Microsoft Word

StatTag inserts objects in Microsoft Word as follows: values are inserted as Word fields; figures are inserted as images; tables are inserted as tables with Word fields in each cell; and verbatim output is inserted as text boxes. Attributes, which are hidden to the user but available programmatically through the Word API, provide the link between the object in Word (Word field, image, table, or text box) and the corresponding tag (value, figure, table, or verbatim).

StatTag also adds a “StatTag version” variable, along with other metadata, to the Word document. The “StatTag version” is not displayed to the end user, but ensures compatibility by allowing StatTag to track the version of StatTag used to update a Word document. Additional information regarding the document metadata is available at: <https://github.com/StatTag/stattag-documentation/blob/master/DocumentMetadata.md>

# S3. Compatibility and Software Updates

StatTag runs R, Python, SAS and Stata code in “batch mode;” code that runs in the program should also work with StatTag. We designed StatTag to minimize changes to routine coding practices. Although code may be modified using StatTag within Word, StatTag is not designed as a primary code editor.

As new versions of the supported statistical programs are released, StatTag’s development team runs unit and integration tests and as necessary develops and releases new versions of StatTag as quickly as possible. The development team does not have access to new versions of statistical software prior to release, and therefore cannot release new versions of StatTag simultaneously. StatTag has worked seamlessly with several software updates (e.g. Stata 15 to Stata 16, most R versions). However, changes to underlying data structures in R 3.5 necessitated substantial changes to StatTag and the associated R.NET (<https://github.com/rdotnet/rdotnet/issues/70>) and RCocoa libraries it uses.

The development team’s process is described within GitHub: <https://github.com/stattag/stattag-documentation#table-of-contents>. Each project has a suite of unit tests that demonstrate correct functionality. The developers run the test suites as part of the development and release preparation workflow. The development team relies on feedback from users, which has substantially improved StatTag over time. Examples of user-contributed enhancement requests are listed in **Supplemental Table 1.**

Supplemental Table 1. User-contributed enhancement requests for StatTag and the associated release.

| **User Request(s)** | **Release (Platform)** |
| --- | --- |
| Improve how StatTag looks on high resolution (high DPI) displays. | V6.0 (Windows) |
| More informative error messages when there is an error in R code. | V3.0.3 (macOS) |
| More streamlined user interface for how StatTag manages creating, editing, and running tags.  Provide a more consolidated “tag manager” view.  Allow tag “placeholders” to make the process of adding multiple tags into a document at once more efficient. | V4.0 (Windows) |
| Notify the user when a linked code file has been changed outside of StatTag. | V3.2 (Windows) |
